# Patient-individual cancer cell lines and tissue analysis delivers no evidence of sequences from DNA viruses in colorectal cancer cells

**DOI:** 10.1186/s12876-020-01404-x

**Published:** 2020-08-06

**Authors:** Michael Gock, Marcel Kordt, Stephanie Matschos, Christina S. Mullins, Michael Linnebacher

**Affiliations:** 1 Department of General Surgery, University of Rostock, Rostock, Germany; 2 Department of General Surgery, Molecular Oncology and Immunotherapy, University of Rostock, Schillingallee 35, D-18057 Rostock, Germany

**Keywords:** Colorectal carcinoma, Oncogenic virus, JC virus, BK virus, Epstein-Barr-virus, Patient-derived carcinoma cell lines

## Abstract

**Background:**

Several DNA viruses are highly suspicious to have oncogenic effects in humans. This study investigates the presence of potentially oncogenic viruses such as SV40, JCV, BKV and EBV in patient-derived colorectal carcinoma (CRC) cells typifying all molecular subtypes of CRC.

**Methods:**

Sample material (gDNA and cDNA) of a total of 49 patient-individual CRC cell lines and corresponding primary material from 11 patients, including normal, tumor-derived and metastasis-derived tissue were analyzed for sequences of SV40, JVC, BKV and EBV using endpoint PCR. In addition, the susceptibility of CRC cells to JCV and BKV was examined using a long-term cultivation approach of patient-individual cells in the presence of viruses.

**Results:**

No virus-specific sequences could be detected in all specimens. Likewise, no morphological changes were observed and no evidence for viral infection or integration could be provided after long term CRC cell cultivation in presence of viral particles.

**Conclusions:**

In summary, the presented data suggest that there is no direct correlation between tumorigenesis and viral load and consequently no evidence for a functional role of the DNA viruses included into this analysis in CRC development.

## Background

The findings gathered in recent years on the relationship between tumorigenesis and several microorganisms, especially viruses, allow a new approach in the prevention and therapy of tumors [1]. For cervical carcinoma associated with human papilloma viruses (especially types 16 and 18, approximately 70% of all cases), there are already worldwide preventive measures and vaccines against the HPV high-risk types in place [2]. If there was a causal relationship between the development of colorectal cancer (CRC) and a viral infection, it would be possible to create new therapeutic and preventive approaches. In 2012 alone, nearly 1.4 million people worldwide developed CRC and 694,000 died as a result. This makes CRC the third leading cause of cancer and the fourth most common cause of cancer death worldwide [3]. In Germany, CRC is the third most common form of cancer among men and the second most among women and lifetime risk to develop a CRC is 5 to 7% [4].

The first oncogenic viruses were discovered in chicken in 1911. Peyton Rous of the Rockefeller Institute was able to isolate a retrovirus (subsequently named after him Rous sarcoma virus) from tumors of diseased chicken and he could demonstrate their malignant potential by causing the same sarcomas after injecting viral particles into healthy chicken. Rous sarcoma virus belongs to the same family of Retroviridae as avian leukosis virus, which is able to cause leukemia in chicken. Later, it could be shown that murine polyomavirus and simian virus 40 (SV40) could cause tumors in experimental animals. SV40 also has a carcinogenic effect on human cells [1]. It is well known that at least 6 different viruses could cause cancer in humans [1]. For the year 2002 it was estimated, that approximately 10–15% of all cancers worldwide were caused by viral infections [5].

Polyomaviruses are icosahedral, nonenveloped viruses with a size of 45 nm. The capsid is composed of 72 pentameric capsomers and surrounds a circular, double-stranded viral DNA genome of approximately 5.5kbp. The virus particles are taken up by endocytosis into the target cell and transported there to the nucleus [6]. It has been repeatedly confirmed that SV40 is oncogenic both in vitro and in vivo [7]. Also for the human polyomaviruses JC virus (JCV) and BK virus (BKV), direct carcinogenic effects are still under debate [8]. Regarding JCV, several case control studies have been conducted to establish the association between JCV seropositivity and specific human tumor types, including CRC [9–15]. BK virus DNA has been detected in several human cancers, including cancer of the head and neck, lung, urogenital tract, liver and pancreas [16, 17].

Epstein-Barr-Virus (EBV) is one of the human pathogenic viruses of the family Herpesviridae. The virus particles are enveloped and have a genome of double-stranded DNA. Seroprevalence in adulthood is over 98%. Though, a direct connection between EBV infection and CRC has not yet been established [18].

The aim of our study was to investigate the presence of potentially oncogenic viruses such as SV40, JCV, BKV and EBV in patient-specific CRC cells, selected to represent all molecular subtypes of CRC. Furthermore, the susceptibility of low-passage, patient-derived CRC cells to the human polyomaviruses JCV and BKV was investigated.

## Methods

### Tissue sampling, cell line establishment and cell culture

Primary CRC resection specimens, specimens of metastases and normal tissue were received fresh from surgery, with informed written patient consent. Tissues were immediately vitally frozen in freezing medium (fetal calf serum (FCS) containing 10% DMSO) at − 80 °C for subsequent examinations. All cell lines were established from patient derived tumor material as previously described by [19] (used cell lines are listed in Table 1). Cell lines were cultured in T75 culture flasks using Dulbecco’s Modified Eagle’s Medium (DMEM)/Ham’s F12 medium supplement with 10% FCS and 2 mM L-glutamine as described before [20]. All procedures involving patient material were approved by the Ethics Committee of the Medical faculty, University of Rostock (reference number II HV 43/2004) in accordance with generally accepted guidelines for the use of human material.

**Table 1:** 49 cell lines were analyzed. Seven cell lines were present in different passages. Of six cell lines only cDNA was analyzed, instead of gDNA and cDNA

Number	Name	Material	Number	Name	Material
1	HROC18 P27	gDNA	29	HROC147Met P28	gDNA
2	HROC24 P31	gDNA	30	HROC147 T0 M1 P34	gDNA
3	HROC24 P42	cDNA	31	HROC183 P9	gDNA
4	HROC24 T1 M2 P53	cDNA	32	HROC183 T0 M2 P23	gDNA
5	HROC24 T3 M1 #2 P9	gDNA	33	HROC212 P17	gDNA
6	HROC32 P34	gDNA	34	HROC222 T1 M2 P8	gDNA
7	HROC32 T0 M2	cDNA	35	HROC257 P10	gDNA
8	HROC32 T3 M1 P10	gDNA	36	HROC257 T0 M1 P9	gDNA
9	HROC39 P30	gDNA	37	HROC257 T0 M1 P54	cDNA
10	HROC39 T0 M1	cDNA	38	HROC277Met2 P8	gDNA
11	HROC40 P29	gDNA	39	HROC277Met2 P9	gDNA
12	HROC46 T0 M1 P40	gDNA	40	HROC277 T0 M1 P43	gDNA
13	HROC50 T1 M5 P29	gDNA	41	HROC278 T0 M1 P10	gDNA
14	HROC57 P31	gDNA	42	HROC278 T0 M1 P24	cDNA
15	HROC60 P38	gDNA	43	HROC278Met P29	gDNA
16	HROC69 P29	gDNA	44	HROC278Met T2 M2 P22	gDNA
17	HROC69 T0 M2 P32	gDNA	45	HROC284Met P28	gDNA
18	HROC80 T1 M1 P25	gDNA	46	HROC285 T0 M2 P29	gDNA
19	HROC87 T0 M2 P25	gDNA	47	HROC296 P15	gDNA
20	HROC87 T0 M2 P29	cDNA	48	HROC296 P16	gDNA
21	HROC103 T0 M1 P36	gDNA	49	HROC324 P10	gDNA
22	HROC107 cT0 M2 P38	gDNA	50	HROC334 P21	gDNA
23	HROC111Met1 T0 M2 P7	gDNA	51	HROC348Met P9	gDNA
24	HROC111Met1 T0 M2 P8	gDNA	52	HROC357 P9	gDNA
25	HROC112Met P30	gDNA	53	HROC357 P14	gDNA
26	HROC113 cT0 M1 P32	gDNA	54	HROC370 P6	gDNA
27	HROC126 P26	gDNA	55	HROC383 P2	gDNA
28	HROC131 T0 M3 P21	gDNA	56	HHC6548 T1 M1 P30	gDNA

Altogether 49 cell lines (Table 1) were tested on Polyomaviridae (SV40, JCV and BKV) and Herpesviridae (EBV). Genomic DNA (gDNA) and complementary DNA (cDNA) were used for the study (all virus preparations were a kind gift of the Institute for Medical Microbiology, Virology and Hygiene, Rostock University Medical Center and serve normally as positive controls in diagnostic virus testing). Seven of the investigated established cell lines were present in different passages (Table 1). Of six cell lines only cDNA was tested (see Table 1 for details). Primary tissue material, including normal, tumor tissue and metastases of 11 CRC patients was analyzed. Altogether 11 tissue samples of tumor material, 10 tissue samples of normal tissue and 3 tissue samples of metastases were included (Table 2).

**Table 2:** Used primary material (*N* = normal epithelial tissue, Tu = tumor tissue, Met = metastasis)

	Tissue type		Tissue type
HROC32	N	HROC212	N, Tu
HROC103	N, Tu	HROC222	N, Tu
HROC107	N, Tu	HROC277	N, Tu, Met
HROC126	N, Tu	HROC278	N, Tu, Met
HROC147	N, Tu, Met	HROC296	N, Tu
HROC183	N, Tu		

### gDNA and RNA isolation

gDNA was isolated from cell pellets using the “Wizard Genomic DNA Purification Kit” from Promega according to the manufacturer’s instructions. The RNA was isolated from cell pellets using the “GeneMATRIX Universal RNA Purification Kit” from EURx according to the manufacturer’s instructions. The measurement of the isolated nucleic acids was then carried out on NanoDrop 1000 from Thermo Scientific.

### cDNA synthesis

cDNA was synthesized from RNA using the Reverase Kit (M-MuLV, RNase H minus) from Bioron (Römerberg, Germany) according to the manufacturer’s instructions. The final cDNA concentration was 12.5 ng/μl after synthesis.

### RT-PCR for SV40 and JC/BK

Primers used for SV40 bind to the specific SV40 nucleotide sequence for the large T antigen (PCR product size: 172 bp). Used primers for JC/BK virus (Table 3) bind to the common specific JC/BK nucleotide sequence for the large T antigen (PCR product size JC/BK: 178 bp and 181 bp). As a positive control for SV40 gDNA, served the SV40 immortalized fibroblast cell line LCT 7. For JC/BK a positive control in form of material from an interlaboratory comparison test with proven high viral load and provided by the Institute of Medical Microbiology, Virology and Hygiene from the Rostock University Medical Center was included. 50 ng of the sample were used as start material for the PCR. Cycling conditions were as follows: 94 °C – 1 min followed by 40 cycles of 94 °C – 30 s, 56 °C – 30 s and 72 °C – 1 min with a final extension at 72 °C for 5 min.

**Table 3:** Primer sequences for SV40 and JC/ BK virus

SV40 largeT forward 5′-AGA TTC CAA CCT ATG GAA CTG A-3**´**	Eurofins MWG Operon LLC; Kentucky Louiseville, USA
SV40 largeT reverse 5′-GAA AGT CCT TGG GGT CTT CTA CC-3´	Eurofins MWG Operon LLC; Kentucky Louiseville, USA
JC/ BK forward 5′-AGG TGC CAA CCT ATG GAA CAG A-3´	Metabion International AG, Planegg, Germany
JC/ BK reverse 5′-GAA AGT CTT TAG GGT CTT CTA CC-3´	Metabion International AG, Planegg, Germany

### RT-PCR for EBV

Primers used for EBV (Table 4) bind to specific EBV nucleotide sequence (PCR product size: 153 bp). As a positive control for EBV gDNA served the EBV immortalized B cell line HROC212. PCR conditions were as described before.

**Table 4:** Primer sequences for EBV

EBV1 forward 5′- CAC TTT AGA GCT CTG GAG GA-3′	Thermo Scientific; USA, Massachusetts
EBV2 reverse 5′-TAA AGA TAG CAG CAG CGC AG-3	Thermo Scientific; USA, Massachusetts

### RT-PCR for β-actin

ß-actin was used as an unregulated household gene to verify that samples are gDNA free after RNA-isolation (PCR product size: cDNA: 210 bp, gDNA: 700 bp). gDNA of cell line HROC111Met1 T0 M2 served as positive control for ß-actin. The annealing temperature was 58 °C. PCR conditions were as described before.

### Sanger sequencing

The initial PCR product was isolated using the „High Pure PCR Product Purification Kit “from Merck (Darmstadt, Germany) according to the manufacturer’s protocol. Next, 50 ng of the isolated product were used as start material for the sequencing PCR. Cycling conditions were as follows: 94 °C – 2 min followed by 35 cycles of 94 °C – 10 s, 62 °C – 20 s and 72 °C – 1 min with a final extension at 72 °C for 5 min. The sequencing reaction contained 5 μl of this PCR product, 2.5 μl nuclease-free water and 2.5 μl primer. They were sent to a commercial sequence service provider (Eurofins, Ebersberg, Germany) which delivered sequence data back.

### Agarose gel electrophoresis

To illustrate fragment sizes, a 100 bp DNA ladder (Carl Roth) was used. For electrophoresis, a voltage of 80 V (15 min) and 100 V (45 min, Elektrophoresis Power Supply, Biometra) was set. Detection took place on the BIO-Print ST4 from VILBER. Separation was performed with 2% Agarose gels in TE-buffer (reagents from Merck).

### Viral infection of cell lines

To test the susceptibility of CRC cells to the JC and BK virus, HROC257 cells were infected with these viruses. The ratio of virus particles and target cells was calculated on the basis of the MOI (= multiplicity of infection) according to the following formula:

$$ \mathrm{MOI}=\frac{\mathrm{Volume}\ \left(\mathrm{Virus}\right)\times \mathrm{concentration}\ \left(\mathrm{Virus}\right)}{\mathrm{Volume}\ \left(\mathrm{cell}\ \mathrm{culture}\right)\times \mathrm{concentration}\ \left(\mathrm{cell}\ \mathrm{culture}\right)}=\frac{\mathrm{Vv}\times \mathrm{Cv}}{\mathrm{Vc}\times \mathrm{Cc}} $$

MOI calculation for JC and BK virus:

$$ \mathrm{MOI}=\frac{40{\mathrm{nm}}^3\times 2,45\times 1{0}^4\mathrm{copies}/\mathrm{ml}}{10\upmu {\mathrm{m}}^3\times 2,50\times 1{0}^2\mathrm{cells}/\mathrm{ml}}=0.39 $$

500 HROC257 cells in 2 ml of DMEM were seeded on a 6-well plate and infected with virus at an MOI of 0.39. One well served as negative control. Cells were subsequently incubated at 37 °C and 5% CO_2_.

## Results

Primary aim of this study was the detection of the potentially oncogenic viruses SV40, JCV, BKV and EBV in patient-individual low passage (<P50) CRC cell lines [21]. Therefore, gDNA and cDNA from established and characterized cell lines and corresponding patient tumor (Tu) and normal (N) tissues, as well as metastases (Met) were examined by RT-PCR.

### Analysis of SV40 in cell lines and primary tissue samples

**Fig. 1:**
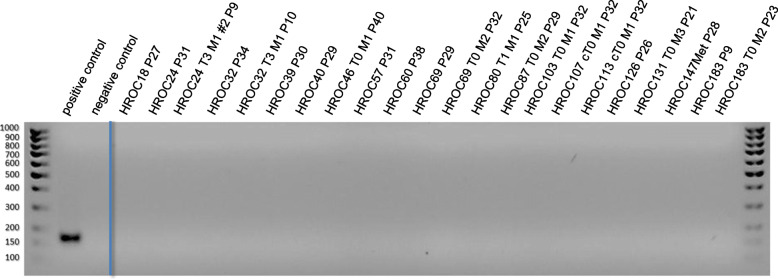
Detection of SV40 by endpoint PCR. Shown are exemplary results of examined cell lines. Material: gDNA, product size 172 bp

For the detection of SV40, altogether 49 individual patient-derived colorectal tumor cell lines were examined. No specific band pattern could be shown for the SV40 virus in all cell lines (Fig. 1).

**Fig. 2:**
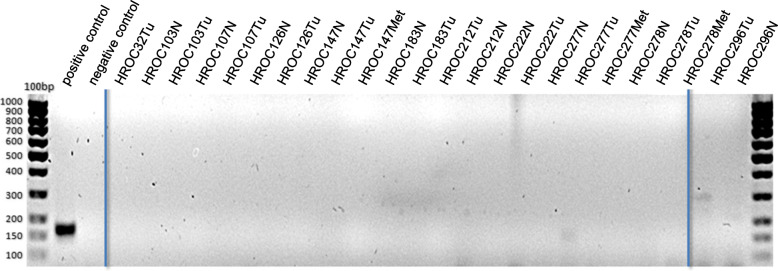
Detection of SV40 by endpoint PCR. Shown are the results of the analysis of primary material. N = normal tissue, Tu = tumor tissue, Met = metastasis. Material: gDNA, product size 172 bp

The positive control shows a clear specific band at 172 bp. In order to clarify whether SV40 sequences are detectable at RNA level, additionally cDNA from the same cell lines was examined. Again, no viral sequences could be amplified with the positive control showing a band at 172 bp (not shown). However, examination of primary tumor material revealed signals in HROC277Tu and HROC296Tu (Fig. 2).

**Fig. 3:**
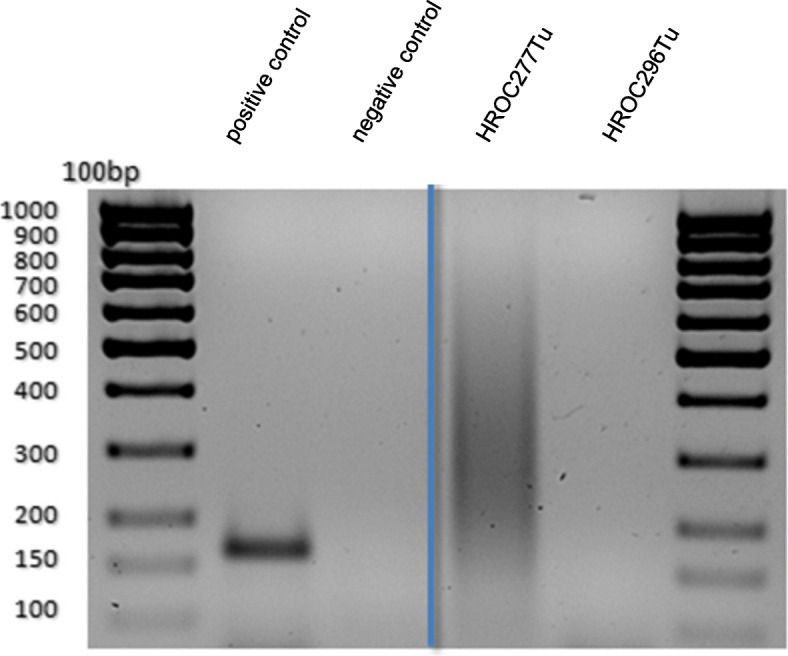
Reamplification PCR for detection of SV40. Material: PCR product of the primary material HROC277Tu and HROC296Tu. Product size of the specific amplicon is 172 bp

PCR product sizes were about 170 bp and 300 bp, respectively. To clarify the specificity of the detected bands, a reamplification of the PCR products was carried out with the same primers. However, the initially detected band of HROC296 (300 bp) could not be reamplified, while the reamplification product of HROC277 exhibited an unspecific smear and must therefore also be regarded as negative (Fig. 3).

### Analysis of JC and BK virus in cell lines and primary tissue samples

**Fig. 4:**
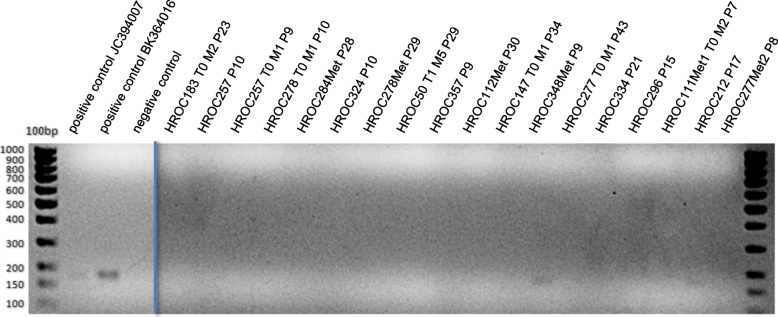
Detection of JC and BK virus by endpoint PCR. Shown are exemplary results of investigated cell lines. Material: gDNA, product size is 178 bp (JCV) and 181 bp (BKV)

**Fig. 5:**
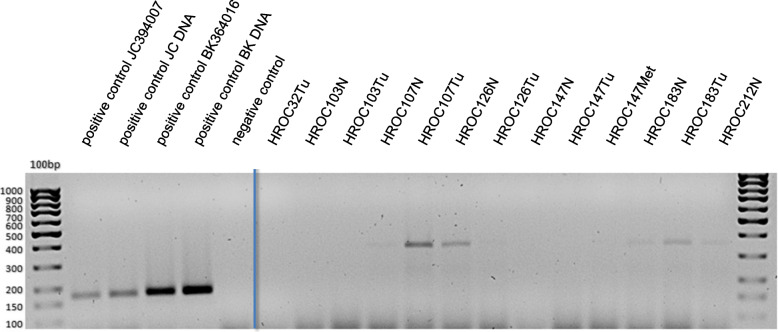
Detection of JC and BK by endpoint PCR. Shown are the results of the analysis of primary material. Material: gDNA, product size is 178 bp (JCV) and 181 bp (BKV)

In 2 of 49 examined cell lines, the PCR with primers designed for the amplification of JCV/BKV sequences delivered a signal at approximately 180 bp (HROC348Met P9 and HROC212 P17) (Fig. 4) suggesting a viral infection. Again, the positive controls delivered the expected products of 178 bp (JCV) and 181 bp (BKV), respectively (Figs. 4 and 5).

To verify these findings, a highly sensitive diagnostic qPCR (TaqMan®) was performed to detect JCV and BKV at the Institute of Microbiology of the University Medical Center, Rostock as described originally by Watzinger et al., in 2004 with minor modifications [22]. However, no JC or BK viral load could be detected in any of the samples and the initial results could thus not be confirmed. In analogy to the SV40 analysis, cDNA was also assayed for JCV and BKV. All cDNA samples were negative (data not shown).

The examination of primary tumor material revealed a signal at approximately 380 bp for the samples HROC107 N/Tu, HROC126 N, HROC183 N/Tu and HROC212 N (Fig. 5). As product size was greater than the expected 178 bp or 181 bp for JC/BK virus, a reamplification PCR was performed with the PCR product of the primary materials. Here, the result of the first RT-PCR could be confirmed. All samples of the reamplification showed a product size of approximately 380 bp. To check the specificity of this amplified nucleotide sequence of the primary patient materials, the reamplified amplicons were sequenced by Sanger sequencing. This analysis identified a human DNA sequence from clone RP11-203 L2 (chromosomes 9q21.11–21.2) with a 99% identification probability. Thus, in none of the investigated primary materials, JCV or BKV were detectable.

### Analysis of EBV in cell lines and primary tissue samples

RT-PCR revealed several amplification products at about 700 bp in size in the examined cell lines and primary tissue samples with no difference between tumor and normal tissue (data not shown). The product size is about 700 bp and is thus by far larger than the expected product size of 150 bp. A reamplification failed to reproduce the initial 700 bp bands in all cases. Similar to the observation for SV40, either an unspecific smear or no signal at all was obtained. In conclusion, it can be assumed that the 700 bp amplicons are non-specific.

### Susceptibility of HROC257 to JC and BK virus

**Fig. 6:**
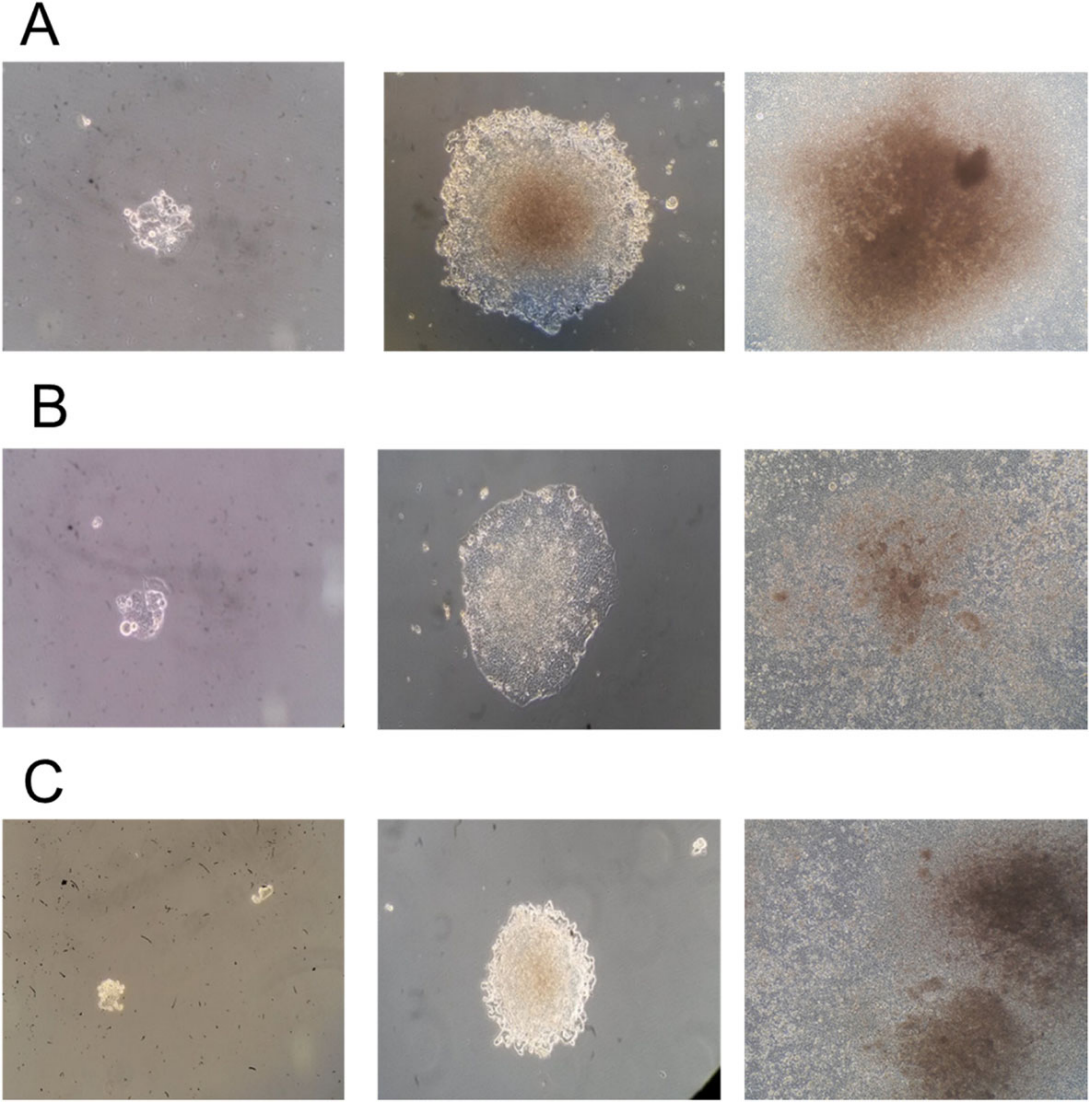
A: Cultivation of HROC257 cells with BK virus after 10, 25 and 38 days of incubation. B: Cultivation of HROC257 cells with JC virus after 10, 25 and 38 days of incubation. C: Negative control (untreated HROC257 cells) after 10, 25 and 38 days. 10x magnification for all cell culture images

To test the susceptibility of CRC cells to infection with JC and BK virus, the cell line HROC257 was infected with JC and BK viruses with an MOI of 0.39 as explained before. Growth and morphology of the cells were documented photographically (Fig. 6). Particular attention was paid to the following parameters: changes in growth behavior / inhibition of proliferation, cell lysis or potential transformatory effects, reflected in the morphology of the cells. During cultivation of the HROC257 cells in the presence of the JCV and BKV, no morphological changes could be observed in comparison to untreated control cells. As shown in Fig. 6, the growth behavior of infected and uninfected cells is similar. In order to further analyze susceptibility to JCV and BKV, these HROC257 cells were subjected to the above described RT-PCR, 40 days after viral infection. Both, cell culture supernatant and isolated cellular gDNA were used as templates. In positive controls, the analysis of cell culture supernatant showed bands at 178 bp (JCV) and 181 bp (BKV). But no products were detectable in supernatant of neither JCV nor BKV treated cells (data not shown). This result suggests that either no or very few viral particles were present in the supernatant. In addition, isolated cellular gDNA of virus-treated HROC257 cells were tested for JCV and BKV after 40 days of incubation. But again no viral amplicon could be detected (data not shown). These results indicate that HROC257 were not susceptible to JC or BK virus infection.

## Discussion

Recent findings shed light into the relationship between tumor development and infections by several viruses. This culminates in the development of preventive vaccinations against cervical carcinoma associated with HPV high-risk types [2]. A causal relationship between the development of CRC and viral infections has repeatedly been suggested and this could, if proved, open new avenues for similar therapeutic and preventive approaches.

Our study aimed at deciphering a potential involvement of several oncogenic DNA viruses in CRC development. The starting hypothesis was that if viruses would have an active role in the development of CRC, their footsteps in form of virus-encoded nucleotide sequences should be present in patients’ CRC cells. In order to avoid any bias by non-tumor cells present in the tumor microenvironment, we focused on patient-individual, low passage CRC cell lines; but corresponding patient tissue samples were analyzed in addition. Using highly sensitive PCR approaches, we investigated the natural occurrence of the polyomaviruses SV40, JCV and BKV as well as the herpesvirus EBV – the most frequently discussed viruses with a potential role in CRC development [9, 10, 13, 15, 21, 23–26]. First, both patient-individual CRC cell lines (*n* = 49) and corresponding tissue samples from patients (normal and tumor tissue, as well as liver, lung and peritoneal metastases) were analyzed for the presence of viral sequences by end-point PCR. But in none of the tested samples, viral sequence amplification was successful.

Regarding a potential SV40 infection, none of the 49 low-passage patient-derived cell lines was positively tested for SV40. This finding excludes SV40 infection at least as a frequent event in CRC cells. It is well known that SV40 might act as a cofactor in the carcinogenesis of several carcinomas; but its involvement in CRC oncogenesis has been examined poorly up to now [27]. Only one case control study by Campello et al. analyzed the involvement of SV40 in CRC pathogenesis [9]. However, only six out of 94 analyzed patients’ tumors were tested positive for SV40 and the authors could not delineate a relationship between viral infection and CRC. An explanation for the differences to the results of the present study could be the extracellular presence of viral particles – either between the cells, more likely in infiltrating lymphocytes or macrophages, and possibly even in the lumen of the gut.

An infection of the analyzed cell lines with the polyomaviruses JCV and BKV could be ruled out likewise. Hitherto existing data on prevalence of JCV infections in CRC are very heterogeneous. Several small case control studies were reported. A study from Portugal could show a prevalence of 90% JCV infections in samples of CRC lesions [15] and in another study from Greek about 60% of the samples tested positive for JCV [28]. A Chinese study of 137 samples described a rate of infection in CRC of 40.9% [13]. But in contrast, a large American study failed to prove JCV infections in 233 patient samples [29] and a study from Iran described a positive detection rate of only 1.46% (2 of 70 patients) [30]. Our own results are pretty much in line with the latter findings and also indicate that JCV is unlikely involved in CRC development.

Worldwide 90 % of adults have been exposed to BKV. BKV DNA has been detected in several human cancers, including carcinoma of the lung, urogenital tract, liver, head and neck, in rhabdomyosarcoma and in brain tumors [17, 31]. However, reports on detection of BKV DNA in CRC tissues are very scarce. A study by Giuliani and colleagues could detect BKV DNA in 9% out of 66 samples [23] and Casini et al. found BKV DNA in several CRC tissues [10]. Again, in our study population in none of the examined cell lines or tissue samples BKV DNA could be detected.

Finally, all cell lines and primary materials were examined for the presence of EBV. Besides non-specific PCR products, presence of EBV DNA could not be verified. All analyzed samples must therefore be considered as EBV negative. Militello et al. described a very low positive detection frequency for EBV DNA in cancerous and cancer-adjacent mucous samples [32] and a study by Cho et al. could not detect EBV in 274 CRC specimen [33]. In contrast, several studies reported a high EBV DNA positivity of CRC tissues [24–26]. Interestingly, a study by Fiorina et al. demonstrated EBV in 23 of 44 CRC samples but they could prove that this was restricted to latency in the lymphoid infiltrate of the tumors [34]. This latter observation delivers the best explanation for the inconsistencies between the before mentioned results. EBV most effectively infects B cells and rarely other lymphoid cell populations. In cultured epithelial-derived cells with no contaminating lymphocytes left over from the initial culturing process, a cross-contamination of the PCR results can safely be excluded. Of note, such a large panel of patient-individual cell lines with corresponding tissue samples has never been tested before. The primary CRC tissues included into our analysis were obtained from the invasive margins which are especially rich in (vital) tumor cells – but infiltrating T cells and also B cells are also readily detectable [35]. Thus, we cannot exclude sporadic positive results in EBV testing due to EBV-positive lymphocytes present in the tumor microenvironment, if a larger sample set of our primary CRC tissue collection would be analyzed.

Based on the data obtained in this work for SV40, JCV, BKV and EBV, it can be concluded that none of the tested viruses are likely to have an obvious general role in CRC development. Possible reasons for the variation in the detection rate of these viruses in our and several of the aforementioned studies, are beside the lymphocytic contamination of the specimens [36], differences in the detection limit of the applied detection systems but possibly also the genetic background of the different patient populations.

In order to verify the general negative findings on a more functional level, we tested exemplary the susceptibility of the CRC cell line HROC257 T0 M1 to JCV and BKV infection. However, even after an extended cultivation period subsequent to viral inoculation, no evidence for a successful viral infection could be provided. This led us to conclude that CRC cells are not susceptible to JC or BK virus infection and thus further supports the negative screening results.

## Conclusion

In conclusion, the presented data suggest that there is no direct correlation between tumorigenesis and viral load of SV40, JVC, BKV and EBV and consequently no evidence for a functional role of the DNA viruses included into this analysis in CRC development.

## Supplementary information

**Additional file 1.**

## Data Availability

The datasets analyzed during the current study are available from the corresponding author on reasonable request.

## Abbreviations

CRC: Colorectal cancer
HPV: Human papilloma viruses
SV40: Simian virus 40
EBV: Epstein-barr-virus
JCV: JC virus
BKV: BK virus
FCS: Fetal calf serum
MOI: Multiplicity of infection
DMEM: Dulbecco’s Modified Eagle’s Medium

## References

[1] Martin D, Gutkind JS (2008). Human tumor-associated viruses and new insights into the molecular mechanisms of cancer. Oncogene.

[2] Lee SJ, Yang A, Wu TC, Hung CF (2016). Immunotherapy for human papillomavirus associated disease and cervical cancer: review of clinical and translational research. J Gynecol Oncol.

[3] Ferlay J, Soerjomataram I, Dikshit R, Eser S, Mathers C, Rebelo M, Parkin DM, Forman D, Bray F (2015). Cancer incidence and mortality worldwide: sources, methods and major patterns in GLOBOCAN 2012. Int J Cancer.

[4] Kaatsch P, Spix C, Katalinic A, Hentschel S, Luttmann S, Stegmaier C, Caspritz S, Christ M, Ernst A, Folkertsor J, Hansmann J, Klein S, Kranzhöfer K, Kunz B, Manegold K, Penzkofer A, Treml K, Weg-Remers S, Wittenberg K, Baras N, Barnes B, Bertz J (2015). Krebs in Deutschland 2011/2012. Gesellschaft der epidemiologischen Krebsregister in Deutschland e.V.

[5] Parkin DM (2005). The global health burden of infection-associated cancers in the year 2002. Int J Cancer.

[6] Ferenczy MW, Marshall LJ, Nelson CD, Atwood WJ, Nath A, Khalili K. Molecular biology, epidemiology, and pathogenesis of progressive multifocal leukoencephalopathy, the JC virus-induced demyelinating disease of the human brain. Clin Microbiol Rev. 2012;25(3):471–506. 10.1128/CMR.05031-11. 10.1128/CMR.05031-11 PMC3416490 22763635

[7] Ahuja D, Sáenz-Robles MT, Pipas JM (2005). SV40 large T-antigen targets multiple cellular pathways to elicit cellular transformation. Oncogene.

[8] Maginnis MS, Atwood WJ. JC virus: an oncogenic virus in animals and humans? Semin Cancer Biol 2009;19(4):261–9. 10.1016/j.semcancer.2009.02.013. 10.1016/j.semcancer.2009.02.013 PMC2694964 19505654

[9] Campello C, Comar M, Zanotta N, Minicozzi A, Rodella L, Poli A (2010). Detection of SV40 in colon cancer: a molecular case–control study from Northeast Italy. J Med Virol.

[10] Casini B, Borgese L, Del Nonno F, Galati G, Izzo L, Caputo M, Perrone Donnorso R, Castelli M, Risuleo G, Visca P. Presence and incidence of DNA sequences of human polyomaviruses BKV and JCV in colorectal tumor tissues. Anticancer Res. 2005;25(2A):1079–85. 15868949

[11] Vilkin A, Ronen Z, Levi Z, Morgenstern S, Halpern M, Niv Y. Presence of JC virus DNA in the tumor tissue and normal mucosa of patients with sporadic colorectal cancer (CRC) or with positive family history and Bethesda criteria. Dig Dis Sci. 2012;57(1):79–84. 10.1007/s10620-011-1855-z. 10.1007/s10620-011-1855-z 21830098

[12] Butcher LD, Garcia M, Arnold M, Ueno H, Goel A, Boland CR. Immune response to JC virus T antigen in patients with and without colorectal neoplasia. Gut Microbes. 2014;5(4):468–75. 10.4161/gmic.29573. 10.4161/gmic.29573 PMC5515464 25007286

[13] Mou X, Chen L, Liu F, Lin J, Diao P, Wang H. Prevalence of JC virus in Chinese patients with colorectal cancer. PLoS One. 2012;7(5):e35900. 10.1371/journal.pone.0035900. 10.1371/journal.pone.0035900 PMC3350510 22606241

[14] Samaka RM, Abd El-Wahed MM, Aiad HA, Kandil MA, Al-Sharaky DR (2013). Does JC virus have a role in the etiology and prognosis of Egyptian colorectal carcinoma?. APMIS.

[15] Coelho TR, Gaspar R, Figueiredo P, Mendonca C, Lazo PA, Almeida L. Human JC polyomavirus in normal colorectal mucosa, hyperplastic polyps, sporadic adenomas, and adenocarcinomas in Portugal. J Med Virol. 2013;85(12):2119–27. 10.1002/jmv.23705. 10.1002/jmv.23705 24009184

[16] Drop B, Strycharz-Dudziak M, Kliszczewska E, Polz-Dacewicz M. Coinfection with Epstein-Barr virus (EBV), human papilloma virus (HPV) and Polyoma BK virus (BKPyV) in laryngeal, Oropharyngeal and Oral cavity Cancer. Int J Mol Sci. 2017;18(12):E2752. 10.3390/ijms18122752. 10.3390/ijms18122752 PMC5751351 29257122

[17] Fioriti D, Videtta M, Mischitelli M, Degener AM, Russo G, Giordano A. The human polyomavirus BK: potential role in cancer. J Cell Physiol. 2005;204(2):402–6. 10.1002/jcp.20300. 10.1002/jcp.20300 15690396

[18] Bedri S, Sultan AA, Alkhalaf M, Al Moustafa AE, Vranic S. Epstein-Barr virus (EBV) status in colorectal cancer: a mini review. Hum Vaccin Immunother. 2018;31. 10.1080/21645515.2018.1543525. 10.1080/21645515.2018.1543525 PMC6605740 30380978

[19] Maletzki C, Stier S, Gruenert U, Gock M, Ostwald C, Prall F, Linnebacher M (2012). Establishment, characterization and chemosensitivity of three mismatch repair deficient cell lines from sporadic and inherited colorectal carcinomas. PLoS One.

[20] Maletzki C, Gock M, Randow M, Klar E, Huehns M, Prall F, Linnebacher M (2015). Establishment and characterization of cell lines from chromosomal instable colorectal cancer. World J Gastroenterol.

[21] Mullins CS, Micheel B, Matschos S, Leuchter M, Bürtin F, Krohn M, Hühns M, Klar E, Prall F, Linnebacher M. Integrated biobanking and tumor model establishment of human colorectal carcinoma provides excellent tools for preclinical research. Cancers (Basel). 2019;11(10). 10.3390/cancers11101520. 10.3390/cancers11101520 PMC6826890 31601052

[22] Watzinger F, Suda M, Preuner S, Baumgartinger R, Ebner K, Baskova L, Niesters HG, Lawitschka A, Lion T (2004). Real time quantitative PCR assays for detection and monitoring of pathogenic human viruses in immunosuppressed pediatric patients. J Clin Microbiol.

[23] Giuliani L, Ronci C, Bonifacio D, Di Bonito L, Favalli C, Perno CF, Syrjänen K, Ciotti M (2008). Detection of oncogenic DNA viruses in colorectal cancer. Anticancer Res.

[24] Liu HX, Ding YQ, Li X, Yao KT (2003). Investigation of Epstein-Barr virus in Chinese colorectal tumors. World J Gastroenterol.

[25] Karpinski P, Myszka A, Ramsey D, Kielan W, Sasiadek MM (2011). Detection of viral DNA sequences in sporadic colorectal cancers in relation to CpG island methylation and methylator phenotype. Tumour Biol.

[26] Rüschoff J, Dietmaier W, Lüttges J, Seitz G, Bocker T, Zirngibl H, Schlegel J, Schackert HK, Jauch KW, Hofstaedter F (1997). Poorly differentiated colonic adenocarcinoma, medullary type: clinical, phenotypic, and molecular characteristics. Am J Pathol.

[27] Vilchez RA, Butel JS (2004). Emergent human pathogen simian virus 40 and its role in cancer. Clin Microbiol Rev.

[28] Theodoropoulos G, Panoussopoulos D, Papaconstantinou I, Gazouli M, Perdiki M, Bramis J, Lazaris AC (2005). Assessment of JC polyoma virus in colon neoplasms. Dis Colon Rectum.

[29] Newcomb PA, Bush AC, Stoner GL, Lampe JW, Potter JD, Bigler J (2004). No evidence of an association of JC virus and colon neoplasia. Cancer Epidemiol Biomark Prev.

[30] Sarvari J, Mahmoudvand S, Pirbonyeh N, Safaei A, Hosseini SY. The very low frequency of Epstein-Barr JC and BK viruses DNA in colorectal Cancer tissues in shiraz, Southwest Iran. Pol J Microbiol. 2018;67(1):73–9. 10.5604/01.3001.0011.6146. 10.5604/01.3001.0011.6146 30015427

[31] Drop B, Strycharz-Dudziak M, Kliszczewska E, Polz-Dacewicz M. Coinfection with Epstein-Barr virus (EBV), human papilloma virus (HPV) and Polyoma BK virus (BKPyV) in laryngeal, Oropharyngeal and Oral cavity Cancer. Int J Mol Sci. 2017;18(12):E2752. 10.3390/ijms18122752. 10.3390/ijms18122752 PMC5751351 29257122

[32] Militello V, Trevisan M, Squarzon L, Biasolo MA, Rugge M, Militello C, Palù G, Barzon L (2009). Investigation on the presence of polyomavirus, herpesvirus, and papillomavirus sequences in colorectal neoplasms and their association with cancer. Int J Cancer.

[33] Cho YJ, Chang MS, Park SH, Kim HS, Kim WH (2001). In situ hybridization of Epstein-Barr virus in tumor cells and tumor-infiltrating lymphocytes of the gastrointestinal tract. Hum Pathol.

[34] Fiorina L, Ricotti M, Vanoli A, Luinetti O, Dallera E, Riboni R, Paolucci S, Brugnatelli S, Paulli M, Pedrazzoli P, Baldanti F, Perfetti V (2014). Systematic analysis of human oncogenic viruses in colon cancer revealed EBV latency in lymphoid infiltrates. Infect Agent Cancer.

[35] Maletzki C, Jahnke A, Ostwald C, Klar E, Prall F, Linnebacher M (2012). Ex-vivo clonally expanded B lymphocytes infiltrating colorectal carcinoma are of mature immunophenotype and produce functional IgG. PLoS One.

[36] Rollison DE (2010). JC virus infection: a cause of colorectal cancer?. J Clin Gastroenterol.

