# Supplementary figures and images for: Patient-individual cancer cell lines and tissue analysis delivers no evidence of sequences from DNA viruses in colorectal cancer cells
Source: BMC Gastroenterol. 2020 Aug 6;20:260. doi: 10.1186/s12876-020-01404-x (PMC7409650; doi:10.1186/s12876-020-01404-x)

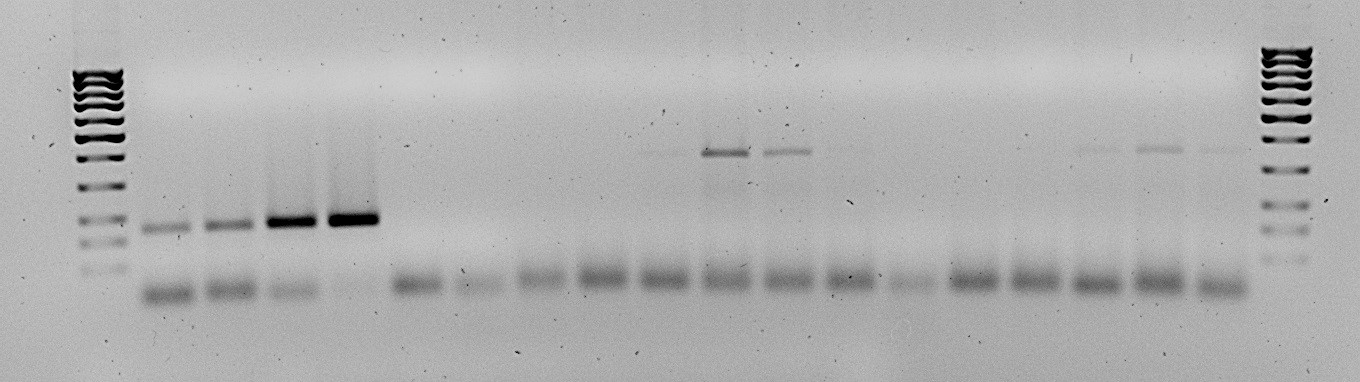

Supplement: Supplementary file 1 — Additional file 1. [file 12876_2020_1404_MOESM1_ESM.zip › Figure 5_uncroppedR3.tif]

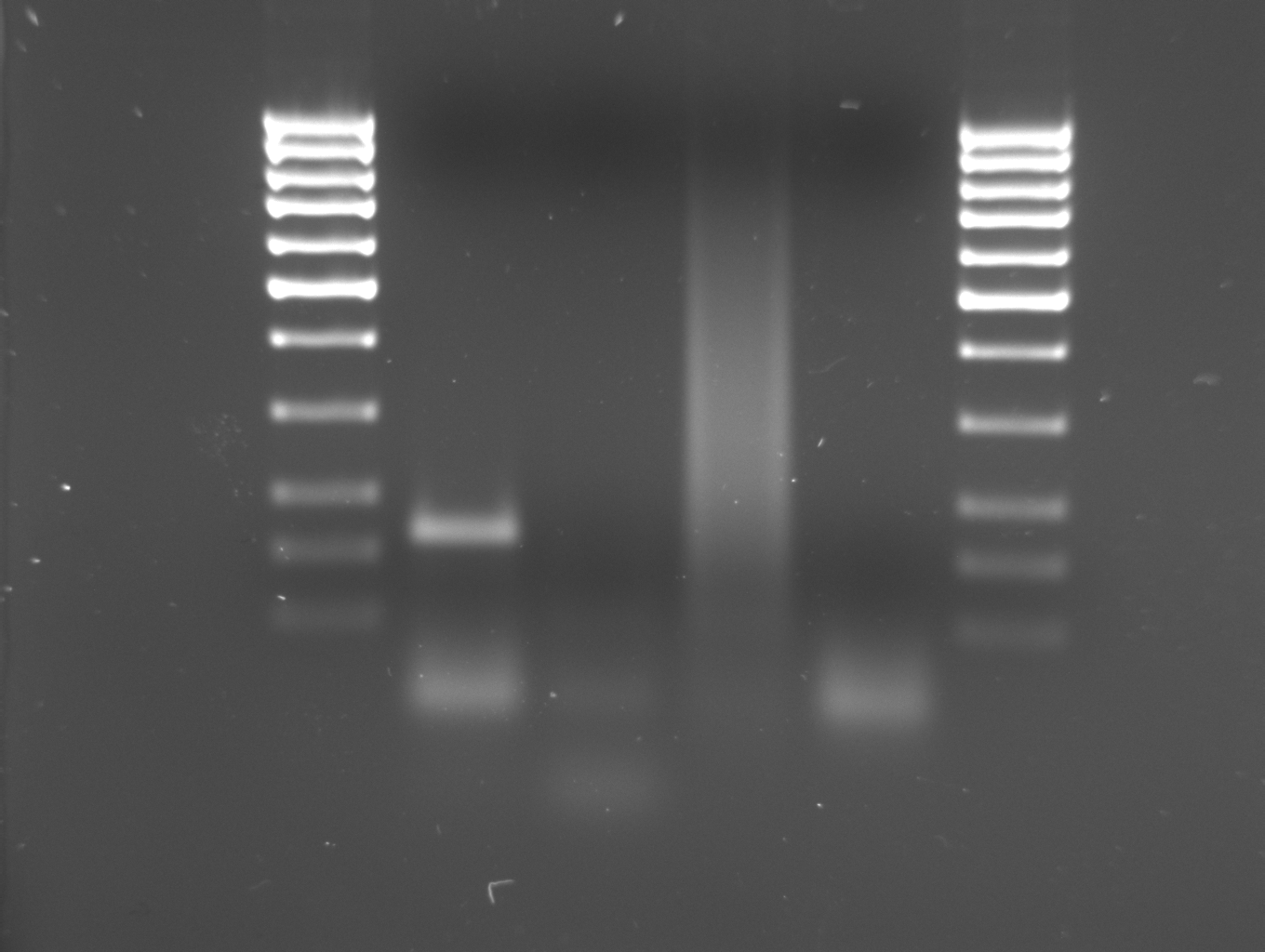

Supplement: Supplementary file 1 — Additional file 1. [file 12876_2020_1404_MOESM1_ESM.zip › Figure 3_uncroppedR3.Tif]

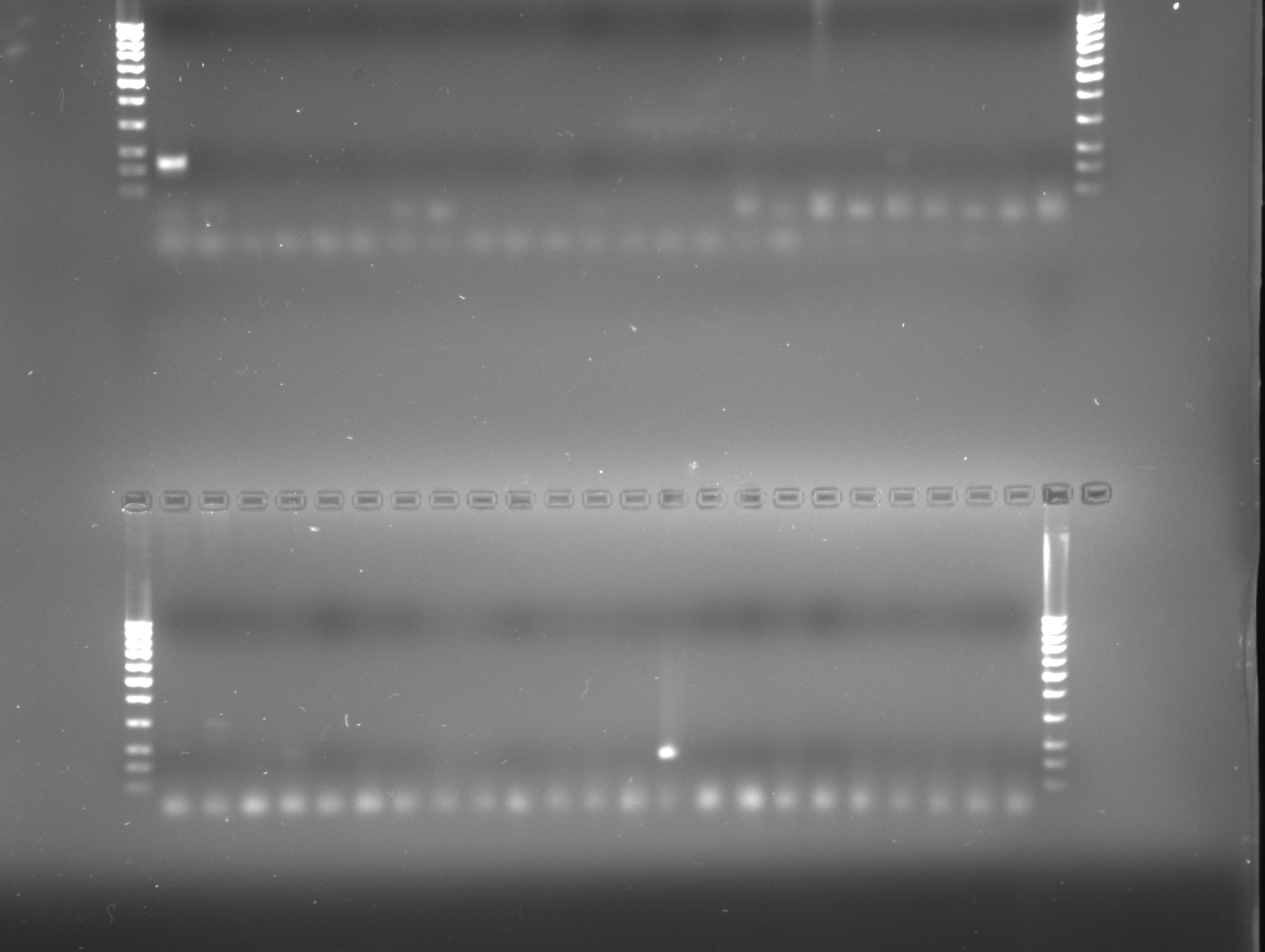

Supplement: Supplementary file 1 — Additional file 1. [file 12876_2020_1404_MOESM1_ESM.zip › Figure 2_uncroppedR3.Tif]

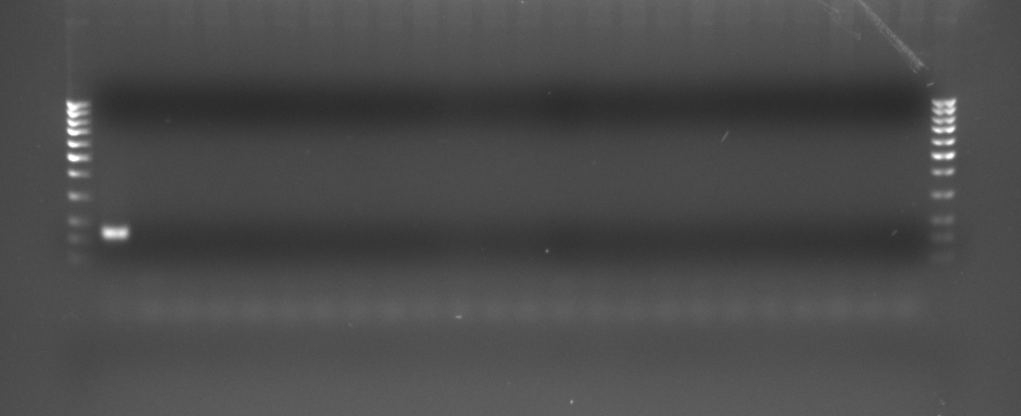

Supplement: Supplementary file 1 — Additional file 1. [file 12876_2020_1404_MOESM1_ESM.zip › Figure 1_uncroppedR3.Tif]
